# Machine‐learning‐derived sleep–wake staging from around‐the‐ear electroencephalogram outperforms manual scoring and actigraphy

**DOI:** 10.1111/jsr.12786

**Published:** 2018-11-13

**Authors:** Kaare B. Mikkelsen, James K. Ebajemito, Maria A. Bonmati‐Carrion, Nayantara Santhi, Victoria L. Revell, Giuseppe Atzori, Ciro della Monica, Stefan Debener, Derk‐Jan Dijk, Annette Sterr, Maarten de Vos

**Affiliations:** ^1^ Institute of Biomedical Engineering University of Oxford Oxford UK; ^2^ Department of Engineering Aarhus University Aarhus Denmark; ^3^ School of Psychology University of Surrey Surrey UK; ^4^ Surrey Sleep Research Centre University of Surrey Surrey UK; ^5^ Surrey Clinical Research Centre University of Surrey Surrey UK; ^6^ Cluster of Excellence Hearing4All Oldenburg Germany; ^7^ Department of Psychology University of Oldenburg Oldenburg Germany

**Keywords:** automated sleep scoring, ear EEG, EEG, home EEG, mobile EEG

## Abstract

Quantification of sleep is important for the diagnosis of sleep disorders and sleep research. However, the only widely accepted method to obtain sleep staging is by visual analysis of polysomnography (PSG), which is expensive and time consuming. Here, we investigate automated sleep scoring based on a low‐cost, mobile electroencephalogram (EEG) platform consisting of a lightweight EEG amplifier combined with flex‐printed cEEGrid electrodes placed around the ear, which can be implemented as a fully self‐applicable sleep system. However, cEEGrid signals have different amplitude characteristics to normal scalp PSG signals, which might be challenging for visual scoring. Therefore, this study evaluates the potential of automatic scoring of cEEGrid signals using a machine learning classifier (“random forests”) and compares its performance with manual scoring of standard PSG. In addition, the automatic scoring of cEEGrid signals is compared with manual annotation of the cEEGrid recording and with simultaneous actigraphy. Acceptable recordings were obtained in 15 healthy volunteers (aged 35 ± 14.3 years) during an extended nocturnal sleep opportunity, which induced disrupted sleep with a large inter‐individual variation in sleep parameters. The results demonstrate that machine‐learning‐based scoring of around‐the‐ear EEG outperforms actigraphy with respect to sleep onset and total sleep time assessments. The automated scoring outperforms human scoring of cEEGrid by standard criteria. The accuracy of machine‐learning‐based automated scoring of cEEGrid sleep recordings compared with manual scoring of standard PSG was satisfactory. The findings show that cEEGrid recordings combined with machine‐learning‐based scoring holds promise for large‐scale sleep studies.

## 1 INTRODUCTION

Sleep is important for general health and disruption of sleep has been associated with poor cognitive performance (Cho, Ennaceur, Cole, & Suh, 2000), metabolic diseases (Garaulet, Ordovás, & Madrid, 2010), cardiovascular diseases (Knutsson & Bøggild, 2011) and overall quality of life. Poor sleep quality has been shown to impact a variety of conditions, such as stroke (e.g. Bassetti, 2005), diabetes, Alzheimer and mental health (e.g. Carr et al., 2018). Therefore, the ability to accurately monitor sleep patterns in the wider population and in the home environment becomes increasingly important.

Recently, significant progress has been made in the field of mobile electroencephalograms (EEGs) (De Vos, Gandras, & Debener, 2014; Debener, Minow, Emkes, Gandras, & de Vos, 2012), indicating that miniaturized EEG systems can be used outside the laboratory environment. An elegant solution to avoid placing electrodes on the head in locations where they are visible or difficult to apply, has been proposed in the form of a miniaturized EEG device placed in or around the ears, offering both a reliable and user‐friendly alternative for full‐scalp EEG (Mikkelsen, Kappel, Mandic, & Kidmose, 2015; Mikkelsen, Kidmose, & Hansen, 2017; Mikkelsen, Villadsen, Otto, & Kidmose, 2017; Pacharra, Debener, & Wascher, 2017). More specifically, several studies have reported progress towards using such ear‐centered EEG devices for tracking the presence of different sleep stages (Looney, Goverdovsky, Rosenzwei, Morrell, & Mandic, 2016; Mikkelsen, Villadsen, et al., 2017; Stochholm, Mikkelsen, & Kidmose, 2016; Zibrandtsen, Kidmose, Otto, Ibsen, & Kjaer, 2016). These studies all showed promising results, but involved a limited number of participants and also restricted electrode positioning. Other user‐mounted systems have been developed specifically for sleep (Levendowski et al., 2017; Younes, Soiferman, Thompson, & Giannouli, 2017 Lucey et al., 2016; Shambroom et al., 2012; Werth and Borbely, 1995), but these systems all require electrodes to be placed in highly visible locations. Ear‐centered EEG solutions come with the benefit of being sufficiently discrete and therefore acceptable to users also for routine applications during the daytime.

In a recent study, we demonstrated that important physiological characteristics can be detected with a lightweight flex‐printed electrode strip that fits neatly behind the ear, the cEEGrid (Debener, Emkes, de Vos, & Bleichner, 2015). Compared with previous ear‐EEG studies, the cEEGrid has the advantage of not requiring individualized electrodes, increased inter‐electrode distances and a larger number of channels. Comparison of the EEG signals obtained from cEEGrid and a standard polysomnography (PSG) montage confirmed the suitability of the cEEGrid for manual sleep staging (Sterr et al., 2018).

Besides the need to reduce manpower for application of the electrodes, there is a growing desire for less time‐consuming manual analysis of sleep recordings. At present, analysis is routinely conducted by time‐consuming visual inspection. When convenient systems such as the cEEGrid become widely available to perform large‐scale sleep monitoring, there will be a radical increase in the number of sleep recordings that need to be annotated. If scoring of such recordings is not automated but continues to depend on manual annotation, the full potential of light‐weight sleep monitoring solutions will not be realized. The present study investigates to what extent a fully automated sleep scoring algorithm can reliably estimate the hypnograms based on the data recorded with cEEGrid electrodes on healthy participants.

Although there is an extensive literature on automated algorithms (for an up‐to‐date review see Boostani, Karimzadeh, & Nami, 2017), we have used ensembles of decision trees, so‐called random forests, on an extended set of features, as this family of classifiers have been shown to perform particularly well for the task of sleep scoring (Boostani et al., 2017; Fraiwan et al. 2012; Mikkelsen, Villadsen, et al., 2017). To investigate the need for specialized algorithms for cEEGrid recordings we compared the performance with a commercial algorithm (packaged with DOMINO by Somnomedics Gmbh) applied to the cEEGrid recordings. Here, comparisons have been performed with sleep parameters and hypnograms obtained with manual scoring as well as sleep–wake assessment derived from actigraphy recordings. We also evaluated different possible cEEGrid channel configurations for automated staging.

## 2 EXPERIMENTAL SET‐UP

### 2.1 Participants

The study was approved by the University of Surrey Ethics Committee. All participants gave written informed consent prior to participation. All data obtained from the study were stored in accordance with the Data Protection Act (1998). Twenty participants, aged 34.9 ± 13.8 years (mean ± *SD*) (eight male) were recruited from the University of Surrey and the general public.

The study participants were asked to stay in bed for 12 hrs between approximately 22:00 and 10:00 hours, during which they were allowed to sleep as much as they wanted (“ad libitum”). Thus, the protocol was designed to induce a recording containing both a substantial amount of wakefulness and sleep, rather than a consolidated sleep episode. This approach provides a more challenging test for automatic sleep scoring. The recordings took place in the sleep laboratory of the Surrey Clinical Research Centre. Each subject slept in a separate sound‐attenuated sleep cabin. Each subject spent only a single night at the centre (i.e. subjects had no adaptation night), thereby increasing sleep disruption. The full study protocol is presented in Sterr et al. (2018).

Two datasets were lost because of human error and three were discarded because of technical problems with either the PSG or the cEEGrid system (i.e. data loss and excessive artefacts). The final sample for analysis comprised 15 participants (six male), aged 35 ± 14.3 years (range, 18−63). They were all fairly good sleepers (Pittsburgh Sleep Quality Index [Buysse, Reynolds, Monk, Berman, & Kupfer, 1989], 2.93 ± 1.71; range, 0–6) and mostly of intermediate chronotype (Morningness–Eveningness Questionnaire [Horne & Ostberg, 1976], 50.4 ± 12.87; range, 15–74).

### 2.2 Recording setup

**Figure 1:**
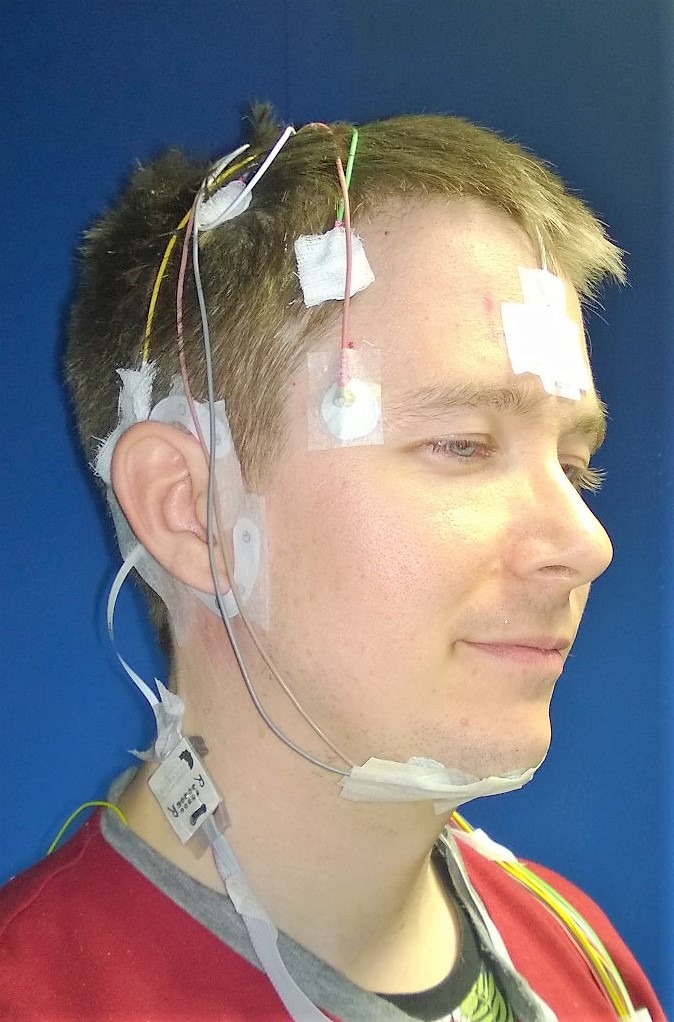
Participant wearing both cEEGrid electrode array and polysomnography electrodes. Permission was obtained from the individual for the publication of this image

Figure 1 illustrates the full sleep recording set‐up. The PSG was recorded at 128 Hz using the SomnoHD system (Somnoscreen SOMNO HD data logger, SOMNOmedics Gmbh, Randersacker, Germany) from six scalp electrodes (F3, F4, O1, O2, C3, C4) referenced to the opposite mastoid (M1, M2) augmented with two ECG leads, two electro‐oculographic (EOG) electrodes and three chin EMG leads (two derivations to one reference). For the EEG and EOG channels, the cut‐off frequencies at—3 dB were 0.3 and 75 Hz, and 0.3 and 110 Hz for the EMG channels.

The Somnomedics system will be referenced simply as “PSG” in the remainder of the paper.

The cEEGrid electrode array consisted of 10 electrodes placed around each ear, labelled as shown in Figure 3(a). During the recording, the electrodes R4a and R4b were used as common ground and reference. Data from the cEEGrid electrodes were recorded with a wireless SMARTING amplifier (mBrainTrain, Belgrade, Serbia) at 250 Hz and a Sony Z1 Android smartphone placed next to the bed. Before the recording began the impedances of all electrodes were measured. If an impedance was larger than 50 kOhm, the electrode was discarded from the analysis; no adjustments to improve impedances were performed. This ensured that the application of the cEEGrid electrodes took <10 min. Participants were given a pre‐programmed Actiwatch (MW8, CamNtech, UK) to be worn on the non‐dominant hand. Data collection started around 22:00 and lasted for about 12 hr. This early and extended sleep opportunity was intended to induce long sleep latencies and low sleep efficiencies, because such sleep periods are more difficult to score and pose a challenge for automatic scoring systems.

## 3 NUMERICAL METHODS

### 3.1 Data preprocessing

Before further analysis, all recordings were imported into EEGLAB v13.5.6b (Delorme & Makeig, 2004) and resampled to 256 Hz, using the “resample” command in Matlab (which uses an anti‐aliasing filter). Afterwards, both PSG and cEEGrid were subjected to 0.5–100‐Hz band pass filtering, and a 50‐Hz notch filter. In effect, as the sampling rate for the PSG recording was only 128 Hz, the upper bound on the pass band was in this case the Nyquist frequency, at 64 Hz.

Precise alignment of the PSG and cEEGrid recordings was achieved by aligning periods of major movement artifacts and/or slow wave activity in both recordings. The details are described in Appendix 1.

High amplitude artifacts were identified using thresholding on signal power calculated in short time windows and were excluded from the analysis. The details of this are described in Appendix 2.

In cases where the cEEGrid recording started after the PSG, the cEEGrid recording was padded with NaN‐values to have identical starting times. However, after both datasets started, the analysis only included epochs for which recordings from both devices were present. This implies that only epochs for which both cEEGrid and PSG data were available were used in the analysis. In two participants, the cEEGrid recording malfunctioned a few hours into the recording. In these cases, only the first part of the night was used in the analysis.

### 3.2 Defining optimal electrode derivations

Because cEEGrid electrode arrays feature a larger number of electrodes than other ear‐EEG systems used for sleep staging, an important question before automated analysis is how to optimally combine them into a few representative derivations. This reduction should maximize both electrode reliability and the amount of information in the derivation. For this, a “correlation index” (CI) was calculated, defined as the correlation of power values within a specific band, weighted by electrode reliability:

(1) $${\text{CI}}_{i}=\sum_{i}\text{corr}\left[P\left(d_{i}\right),P\left(s_{j}\right)\right]g_{i}$$

where *d*_*i*_ is the *i*’ith cEEGrid derivation tested, *s*_*j*_ is the *j*’th scalp derivation, *P* (*h*) is the integrated power of channel *h* over a sleep epoch, and *g*_*i*_ is the fraction of time over the whole dataset in which the cEEGrid derivation has good‐quality data.

The derivations tested are all intra‐C single electrode derivations (two electrodes within the same “C” referenced to each other): the average of one C versus the other (“L–R”), top electrodes versus bottom electrodes (“TB”, defined as channels 2 and 3 versus channels 6 and 7 in each “C”) and front electrodes versus back electrodes (“FB”, defined as electrodes 1 and 8 versus electrodes 4 and 5).

In the analysis we focused on the correlation within specific power bands, specifically alpha (8–16 Hz), beta (16–32 Hz), theta (4–8 Hz) and delta (1–4 Hz).

### 3.3 Sleep scoring

#### 3.3.1 Actigraphy scoring

The actiwatch recording was passed through the algorithm developed by the actiwatch manufacturer (CamNtech). It consisted of partitioning the recording into 60‐s epochs and smoothing the epoch activities, weighting neighbouring epochs by 20% and neighbours by 4%. Finally, the smoothed recording was subjected to a threshold, below which the participant was scored as sleeping. After processing, the 60‐s epochs were transformed into 30‐s epochs, inheriting the score of the parent epoch.

#### 3.3.2 Manual sleep scoring of PSG and cEEGrid

Two experienced sleep technicians scored all recordings, both PSG and cEEGrid. To avoid previous knowledge from the PSG recording influencing the cEEGrid scoring, the cEEGrid recordings were anonymized. Scoring was based on 30‐s non‐overlapping epochs according to the guidelines of the American Association for Sleep Medicine (AASM; Berry et al., 2017). Even though amplitude characteristics of the EEG are different between cEEGrid and traditional EEG derivations, no adjustment of amplitude criteria was implemented for scoring cEEGrid recordings. Preliminary analysis revealed inter‐rater concordance in >98% of epochs. Based on this, we decided to only use the scoring from the first technician in the analyses presented in this manuscript.

In the remainder of the analysis, all non‐scored epochs (meaning that the manual label was either “A” or “artefact”) were removed from both PSG and cEEGrid datasets.

#### 3.3.3 Automatic sleep scoring

For automatic sleep scoring, we developed a custom‐made sleep scoring algorithm (using a “random forest” classifier as described below) by closely following the feature‐based approach proposed in Mikkelsen, Villadsen, et al. (2017) (in turn inspired by Koley & Dey, 2012).

As a benchmark to compare the custom‐made algorithm against, both PSG and cEEGrid recordings were also analyzed using the automatic algorithm packaged with the DOMINO software supplied by Somnomedics Gmbh (Randersacker, Germany). Depending on the quality of the sleep recordings, we should expect the DOMINO software to outperform the random forest classifier for the PSG recordings, while being less ideal for the cEEGrid recordings.

##### Features

We computed the 33 features listed in Table 1 for the three chosen derivations. For automatic sleep staging based on PSG, the chosen channels were EOG1:M2, EMG2:EMG3 and C4:M1. For staging based on cEEGrid, the selected derivations were FB(L), FB(R) and L–R (front versus back for each ear, and the ears relative to each other, shown in Figure 3b). We also simulated the effect of adding an EOG channel to the cEEGrid data, where we combined the original 99 cEEGrid features with 33 features extracted from EOG1:M2. For this particular case, the “channel correlation” feature (F7 in Table 1) was calculated between “EOG1:M2” and “L–R”. Feature selection was investigated (see Appendix 3); however, we found that the possible benefits from reducing the number of features were very limited (an improvement in Cohen's kappa of about 0.05 points), and therefore decided to use all features.

**Table 1:** An overview of the features used in this study, grouped by type. All features are described in Mikkelsen, Villadsen, et al. (2017). The EOG and EMG “proxies” are created by band‐pass filtering the cEEGrid data (using 0.5–30 Hz for the EOG proxy and 32–80 Hz for the EMG proxy)

Label	Short description	Type
F1	Signal skewness	EEG time domain
F2	Signal kurtosis	
F3	Zero crossing rate	
F4	Hjorth mobility	
F5	Hjorth complexity	
F6	75th percentile	
F7	Channel correlation	
F8	EMG power	EMG proxy
F9	Minimal EMG power	
F10	Relative EMG burst amplitude	
F11	Slow eye movement power	EOG proxy
F12	Rapid eye movement power	
F13, F14, F15, F16	Relative power in α*,* β*,* θ*,* δ‐bands	EEG frequency domain
F17, F18, F19, F20, F21, F22	Power‐ratios: α*/*δ*,* δ*/*β*,* δ*/*θ*,* θ*/*α*,* θ*/*β*,* α*/*β	
F23	(θ* *+ δ)*/*(α* *+ β)	
F24	Spectral edge frequency	
F25	Median power frequency	
F26	Mean spectral edge frequency difference	
F27	Peak power frequency	
F28	Spectral entropy	
F29	Spindle probability	Sleep event proxies
F30	Frequency stationarity	
F31	Lowest adj. frequency similarity	
F32	Largest CWT value	
F33	Longest sleep spindle	

EEG, electroencephalogram; EOG, electro‐oculographic. EMG, electromyography; CWT, continuous wavelet transform.

If part of an epoch was discarded, in either one, two or three derivations, the features were calculated based on the remaining part of the epoch. If the entire epoch was rejected, all features were set to “NaN”, and the epoch would be scored as “awake” (because this would usually be a result of large amounts of movement).

##### Random forest classifier

The features were passed to a “random forest” (Breiman, 2001), an ensemble of “decision trees” consisting of 100 trees. The implementation used the “fit ensemble” function in Matlab 2016b, with the “Bag” algorithm. Each tree was trained on a resampling of the original training set with the same number of elements (but duplicates allowed). For each tree, splitting optimized the Gini coefficient (Ceriani & Verme, 2011) and continued until all leaves (subgroups) were homogeneous. Leave‐one‐subject‐out validation was performed to obtain classification results for all subjects. When testing classifiers, we have experimented with different sources of data and training based on different labels. Unless otherwise stated, ground truth was the visual scoring of the PSG. We describe our nomenclature used in Table 2.

**Table 2:** Description of nomenclature used in exploration of different automatic processing experiments

Name	Method
Aut. PSG	Automatic scoring using features derived from polysomnography (PSG) data and training based on the labels from manual PSG
cEEGrid	Automatic scoring using features derived from cEEGrid data and training based on the labels from manual PSG
cEEGrid‐manual	Manual scoring of cEEGrid recording
cEEGrid+EOG	Automatic scoring using features derived from cEEGrid data as well as the electro‐oculographic (EOG) channel from the PSG, as described above. Training labels are obtained from the manual PSG scoring
cEEGrid*	Automatic scoring using features derived from cEEGrid data, using training labels from manual cEEGrid scoring. Ground truth for testing was based on manual annotation of cEEGrid as well. This means that when kappa and accuracy values are computed, cEEGrid‐based hypnograms are used, and not PSG‐based ones
Actiwatch	Automatic scoring using actiwatch software (CamNTech, Cambridge, UK)
PSG DOMINO	Automatic scoring of PSG recording using the DOMINO software from Somnomedics Gmbh
cEEGrid DOMINO	Automatic scoring of cEEGrid recording using the DOMINO software from Somnomedics Gmbh

EMG, electromyography; CWT, continuous wavelet transform.

### 3.4 Hypnogram post‐processing

The classifier described does not consider neighbouring epochs. However, as certain patterns are used during visual analysis, we implemented three steps in a post‐processing phase to increase the plausibility of the estimated hypnograms:

#### 3.4.1 Determine sleep onset

To avoid spurious sleep detections during wake, it was required that sleep onset should be followed by 5 min (10 epochs) of consecutive sleep. This is also known as latency to persistent sleep. Thus, sleep onset was taken as the beginning of the first epoch fulfilling this criterion.

#### 3.4.2 Determine wake up

Wake up had to be preceded by 5 min of sleep, and was taken as the end of the last epoch meeting this criterion.

#### 3.4.3 Smooth hypnogram

For the period between falling asleep and waking up, class probabilities were extracted from the classifier. The probabilities were smoothed with a moving average window of five epochs. For each epoch, the resulting label is the class with the highest smoothed probability. The only exception to this is that all wake epochs are retained to preserve brief mid‐night arousals.

The first two stages of post‐processing were also used on the actigraphy‐based hypnograms, to obtain a fairer comparison.

This post‐processing step was chosen instead of other multi‐epoch approaches (such as those discussed in Phan, Andreotti, Cooray, Chén, & Vos, 2018) because the performance of this solution was very similar, but allows for a relatively simple description.

### 3.5 Sleep statistics

To better quantify the agreement between automatically and manually generated hypnograms, a selection of relevant sleep statistics was calculated. Correlations between whole‐recording sleep statistics derived from automatically scored cEEGrid, cEEGrid + EOG and actigraphy and sleep statistics derived from manually scored PSG were computed. An overview and definition of used sleep statistics is shown in Table 3.

**Table 3:** Overview and description of sleep statistics used in this study

Label	Description
Total sleep	Total duration of all epochs scored as “sleep”
Sleep efficiency	Total sleep divided by duration of recording
Wake after sleep onset (WASO)	Duration of wake epochs between falling asleep and waking up (defined as the end of the last epoch scored as sleep). This means that wake epochs after final awakening were not included in the calculation of this variable
Sleep onset latency	Time from start of recording until first sleep epoch
Rapid eye movement (REM) latency	Time from sleep onset until first REM epoch

## 4 RESULTS

### 4.1 Data quality and choice of derivations

In total, 18 920 epochs were used for the automatic scoring, which corresponds to an average of 10.5 hr per participant (range, 3.1–12.0 hr).

Table 4 shows the percentage of time spent in different stages (percentage of total recording time), as estimated by the different approaches. We note that because the cEEGrid estimate of “wake percentage” is very close to the manual PSG‐based estimate, it is also very good for “pooled sleep” (which is simply everything else). This table also shows that sleep was quite disrupted during the extended sleep opportunity protocol, such that on average participants were awake for 45% of the recording period.

**Table 4:** Percentage of epochs scored as each stage, calculated by pooling all subjects before calculating average (instead of average of subject averages)

	Manual PSG	Aut. PSG	cEEGrid + EOG	cEEGrid	Actiwatch
Wake	34.8%	43.9%	45.1%	38.4%	25.5%
REM	13.0%	09.1%	06.1%	06.7%	–
N1	06.7%	01.5%	0.5%	00.1%	–
N2	34.4%	36.4%	41.0%	47.1%	–
N3	11.1%	9.1%	07.3%	07.7%	–
Pooled sleep	65.2%	56.1%	54.9%	61.6%	74.5%

PSG, polysomnography; EOG, electro‐oculographic; REM, rapid eye movement. EMG, electromyography; CWT, continuous wavelet transform.

**Figure 2:**
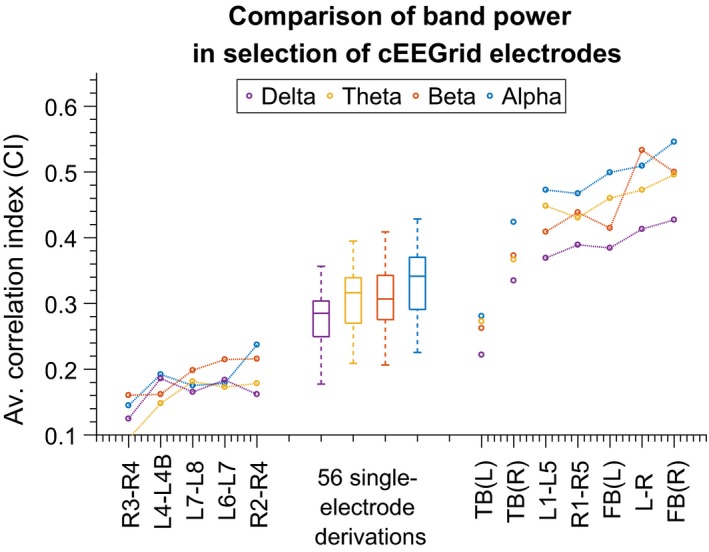
Comparison of electrode derivations. The metric is “CI” as defined in Equation (1). “TB” in this context means “top versus bottom” and FB(L) and FB(R) mean “front versus back”, for left and right ear, respectively. Values for TB(L) and TB(R) have been plotted as “detached” from the rest of the derivations because they have been positioned on the x‐axis outside of their place in the ordering (which would have hidden them inside the group of 56 “single‐electrode” derivations)

**Figure 3:**
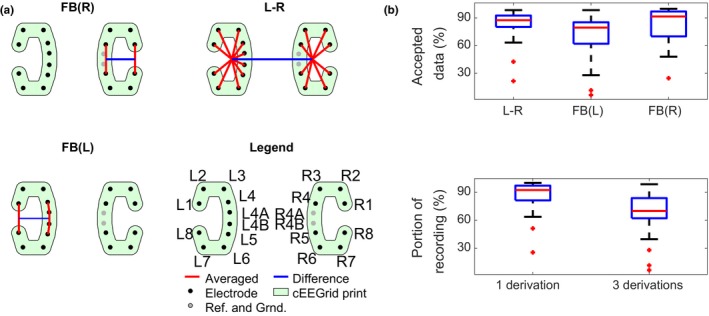
(a) Visualization of the three chosen derivations. FB(L) and FB(R) are within‐C, and L–R is the difference between the average within each C. (b) Analysis of reliability of the three chosen derivations. “Accepted data” is equivalent to *g*_*i*_ in (1). FB(L) and FB(R) mean “front versus back”, for left and right ear, respectively; L–R, average of left electrodes versus average of right electrodes.

Figure 2 shows *CI*_*i*_ (Equation (1)) plotted for four different frequency bands, and a range of electrode combinations. Good correlations were obtained by using electrode averages and larger electrode distances. Based on this, we have chosen to use three standardized derivations: L–R (average of left electrodes versus average of right electrodes), FB(L) and FB(R) (average of front versus average of back electrodes in both “Cs”). This yields a combination of high information content and reliability. The final choices are shown graphically in Figure 3(a).

After artifact rejection of channels and epochs, Figure 3(b) shows the reliability (i.e. how much is left after artifact removal) of the three aggregated derivations. Pooling the electrodes makes them more reliable, and it is rare that no more than one electrode in each group is available.

### 4.2 Automatic sleep scoring

**Figure 4:**
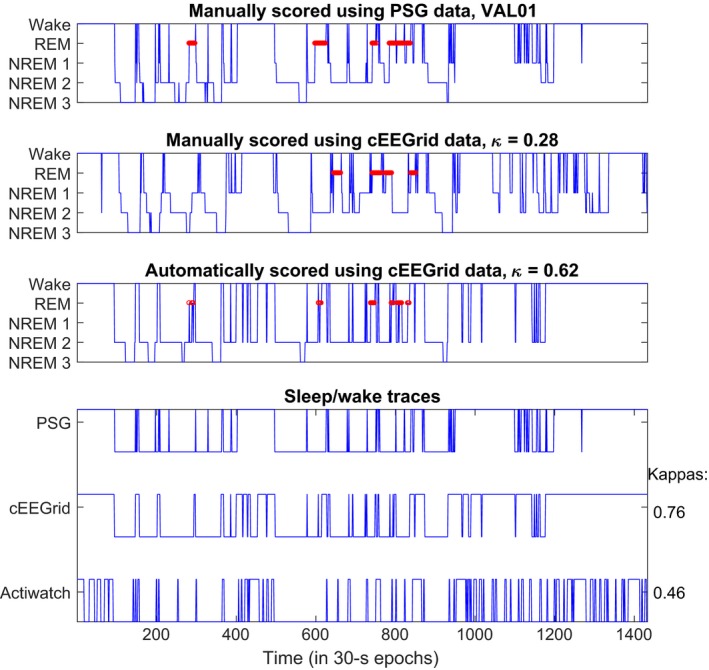
Representative hypnograms for a sleep recording. Top shows the manual scoring, using polysomnography data. Middle shows manual scoring using cEEGrid data and bottom shows automatic scoring after post‐processing, using leave‐one‐subject‐out cross‐validation

Figure 4 illustrates the scoring for one subject. It compares manual annotation based on PSG, and both manual and automatic annotation based on cEEGrid data. We see that the algorithm captures the overall structure of the night's sleep well, with some added short transitions, outperforming visual scoring of the same recording.

**Figure 5:**
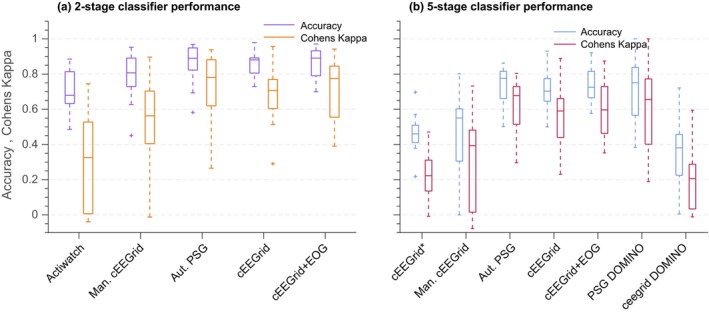
Comparison of classification performance for various automatic sleep staging algorithms and one manual classification. (a) Using only sleep–wake scores, (b) using five‐stage scoring. Labels on the *x*‐axis are described in detail in Table 2

In Figure 5a classification performance is shown for sleep–wake classification based on actigraphy and cEEGrid‐based scoring. We see that average performance increases (for accuracy and Cohen's kappa) when EEG information is incorporated, and automatic cEEGrid scoring is markedly better than the actiwatch scoring.

Figure 5(b) shows the distributions of sleep staging accuracies as well as Cohen's kappa values. Different ways of training the classifier and annotating the data, and the use of different input signals, are compared, as described in Table 2.

Table 5 shows *p*‐values (not corrected for multiple testing) for the null hypothesis that the kappa values derived for cEEGrid and PSG have equal means to the value derived for alternative hypnogram sources. We see that the two worst performing methods, “cEEGrid*” and “actiwatch” are significantly different (worse) than automatic cEEGrid.

**Table 5:** Calculated *p*‐values for the null hypothesis (using a Student's *t* test) when kappa values derived for automatic cEEGrid scoring and those derived for other means of scoring have equal means (as seen in Figures 5 and 6). All *p*‐values are calculated using paired two‐tailed *t* tests

	cEEGrid*	Actiwatch	cEEGrid‐manual	Aut. PSG	cEEGrid + EOG	PSG DOMINO
Two‐stage						
Mean ± *SD*	0.214 ± 0.140	0.246 ± 0.165	0.153 ± 0.331	−0.030 ± 0.182	−0.024 ± 0.145	−0.02 ± 0.204
*p*‐value	0.000	0.000	0.096	0.532	0.534	0.704
Five‐stage						
Mean ± *SD*	0.176 ± 0.115	–	0.151 ± 0.266	−0.043 ± 0.178	−0.036 ± 0.092	−0.065 ± 0.225
*p*‐value	0.000	–	0.045	0.365	0.154	0.286

PSG, polysomnography; EOG, electro‐oculographic.

In particular, as the initial visual scoring of cEEGrid data was not always very accurate, the classifier trained on cEEGrid‐based labels performed worse than that based on PSG‐based labels. This suggests that the to‐be‐expected improvement from sharing information between the human scorer and automatic classifier is more than offset by the reduction in actual brain‐state information contained in the manual labels when switching from PSG‐based to cEEGrid‐based training labels. This also highlights the need to perform simultaneous PSG measurements for use in training automatic classifiers, when testing reduced montages such as the cEEGrid (in other words: a manual scoring of the reduced montage recording cannot substitute as a ground truth for use in algorithm development).

**Figure 6:**
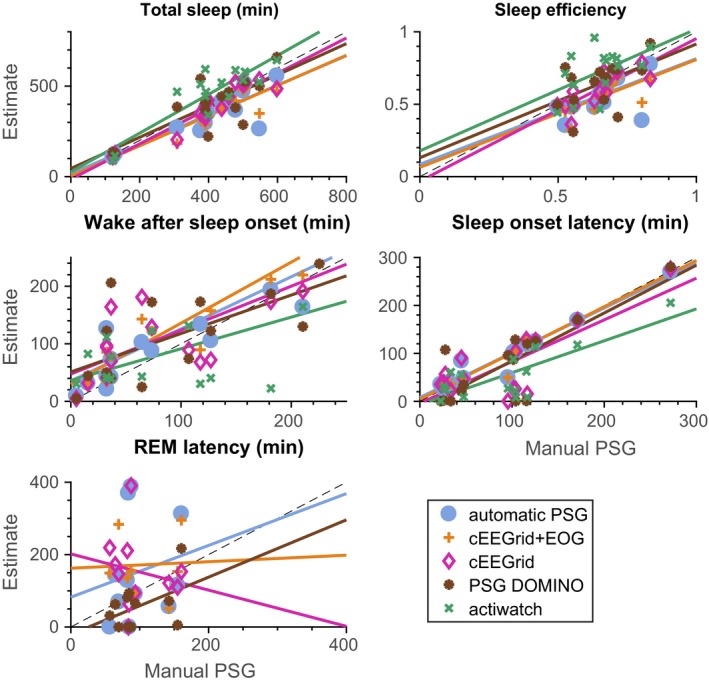
Sleep statistics. See Table 2 for a description of the methods compared. For each plot four *r* ^2^‐values can be calculated, according to how well the straight line fits to the scatter plot. The *r* ^2^‐values for each plot are (in the order of the legend): total sleep (0.72; 0.84; 0.89; 0.61; 0.78), sleep efficiency (0.34; 0.49; 0.65; 0.25; 0.27), WASO (0.55; 0.70; 0.52; 0.42; 0.32), SOL (0.94; 0.96; 0.61; 0.67; 0.63), REM latency (0.04; 0.00; 0.03; 0.21; NaN). WASO, wake‐after‐sleep‐onset; REM, rapid eye movement; SOL, sleep onset latency

Figure 6 compares distribution of a range of sleep statistics for the different types of classifiers described in Table 2. The automatic cEEGrid classifiers generally performed better than visual cEEGrid annotation or actigraphy. For estimating REM periods, adding the EOG helped to identify REM stages. None of the methods reliably captured the short awakenings as annotated in the PSG. This is also reflected in Figure 4.

Table 6 shows *p*‐values for paired *t*‐tests, testing whether the sleep statistics for manual PSG scoring have the same mean values as those of the alternative methods. We see that the fact that *p*‐values are sensitive to bias and not covariance means that primarily the “noisy” estimates have non‐significant *p*‐values, whereas, for instance, “total sleep” as estimated from automatic PSG‐based scoring is significantly different to that estimated from manual PSG scoring, despite an *r* ^2^‐value of 0.72.

**Table 6:** Calculated *p*‐values for the null hypothesis (using a Student's *t* test) that the sleep statistics from automatic scoring are from distributions with the same mean values as those derived from manual , polysomnography scoring (as seen in Figure 7). All *p*‐values are calculated using paired two‐tailed *t* tests

	Aut. PSG	cEEGrid + EOG	cEEGrid	PSG DOMINO	actiwatch
Total sleep	0.008	0.001	0.058	0.672	0.014
Sleep efficiency	0.005	0.000	0.04	0.805	0.053
Wake after sleep onset	0.099	0.016	0.079	0.180	0.887
Sleep onset latency	0.679	0.788	0.202	0.221	0.003
REM latency	0.221	0.127	0.142	0.039	–

PSG, polysomnography; EOG, electro‐oculographic; REM, rapid eye movement.

Table 7 shows intra‐class correlation (ICC) values, comparing the sleep statistics from the methods described in Table 2 with those derived from the manual scorings. We see that the ICC values generally tell the same story as the *r* ^2^‐values, with automated estimates based on either PSG or cEEGrid being comparable, and REM latency generally being estimated very poorly.

**Table 7:** Intraclass correlation coefficients (ICCs) between manual PSG‐based scoring and the alternatives for sleep statistics. The precise type of ICC was “ICC(A,1)”, as described in McGraw and Wong (1996), and implemented in Salarian (2016)

	Aut. PSG	cEEGrid + EOG	cEEGrid	PSG DOMINO	Actiwatch
Total sleep	0.778	0.819	0.933	0.787	0.824
Sleep efficiency	0.435	0.471	0.750	0.464	0.345
Wake after sleep onset	0.698	0.757	0.690	0.633	0.566
Sleep onset latency	0.969	0.979	0.771	0.796	0.662
REM latency	0.087	0.010	−0.101	0.314	–

PSG, polysomnography; EOG, electro‐oculographic; REM, rapid eye movement.

## 5 DISCUSSION AND CONCLUSION

We investigated the performance of automated sleep staging of data recorded from cEEGrid electrodes, comparing it with manual scoring of the same data as well as simultaneously recorded PSG data in a protocol in which sleep was disrupted. This is relevant for sleep monitoring in healthy participants and patients at home, where manual annotation of huge amounts of daily recordings would be unmanageable. The study confirms that EEG from cEEGrid sensors preserves the information needed for reliably scoring sleep in healthy subjects. The automatic sleep staging of cEEGrid data leads to a similar accuracy as automatically staged PSG recordings. Automatic scoring extracts more valid sleep statistics than automatically annotated actigraphy data and provides more accurate hypnograms from cEEGrid recordings than visual annotation and when visual annotation of PSG is taken as the ground truth.

Although the latter result might seem surprising, we consider two explanations. On the one hand, AASM rules for sleep staging have been defined for standardized PSG montages, and might be suboptimal for montages with non‐standardized positions, such as the cEEGrid solution. On the other hand, because of the non‐standardized positions of cEEGrid electrodes, the human annotator may struggle to reliably pick up certain features in a particular visualization. However, because automated sleep staging reliably extracts the relevant information, it is clear that the cEEGrid does pick up sleep‐stage‐sensitive features, despite the alternative positioning. Other mobile EEG solutions intended for sleep assessment will also most likely have different electrode positions to standard PSG, to avoid sites with hair or positions in which the electrodes may be visible. These solutions might thus also benefit from automated scoring and our results support continued research in automated annotation of sleep stages. Related to this, it was harder to algorithmically reproduce cEEGrid‐based manual labels than PSG‐based manual labels when the algorithm only used cEEGrid data. This suggests that even for a very experienced scorer, it is more difficult to score sleep based on non‐standard derivations. This should be kept in mind when new EEG solutions are validated. Such comparisons should not only use manual scoring, as the results might be confounded by the uncertainty from non‐standard electrode locations. As automated algorithms for new EEG systems need to be trained on labelled data, this research suggests collecting data from subjects recorded simultaneously with the new system and traditional PSG, so that PSG‐based labels can be used for training the algorithm.

When manual scoring is still preferred (this could be the case in relation to certain sleep disorders), we highly recommend cEEGrid‐specific training of the sleep technician.

Additionally, we compared the performance of our automatic classifier with that of a commercial system (DOMINO). We observed a similar performance on PSG data and, not surprisingly, markedly worse performance when the commercial system was applied to cEEGrid data. That the commercial system achieved a less than ideal score may be because it was not developed to score other than standard signals or it does not perform well scoring disrupted sleep patterns, such as those created by the current protocol.

Another question addressed is how the redundancy of electrodes on cEEGrid can be exploited to increase the reliability for sleep staging. For this, we examined correlations between cEEGrid data and PSG data, while taking electrode reliability into account. This revealed that the horizontal derivations FB(R) and FB(L) were the most informative for preserving sleep‐relevant information. This is similar to work presented in Bleichner, Mirkovic, and Debener (2017), where the significance of cEEGrid channel orientations for picking up far‐field electrical activity was discussed. In the present study, we exploited the electrode redundancy to obtain a reliable signal representation in all instances during sleep. Optimal placement of electrodes when only a limited number of electrodes are available, is a highly important and under‐documented challenge. In our previous work, we derived optimal low‐density channel positions and orientations from initial high‐density EEG evaluations (Zich et al., 2015). However, this is not feasible in a sleep setting. We anticipate that the present findings will be informative for future around‐the‐ear EEG work.

The hypnograms generated by visual and annotated scoring mainly differ in estimates of wake‐after‐sleep‐onset (WASO), as seen in Figure 6. WASO is an important parameter for evaluating sleep quality, but with the current automated assessment of cEEGrid data cannot yet be reliably assessed. We hypothesize that this is because of the different EEG characteristics between brief arousals and “proper” wake EEG. The WASO‐related problems were also observed in other studies; see Myllymaa et al. (2016), Popovic, Khoo, and Westbrook (2014) and Griessenberger, Heib, Kunz, Hoedlmoser, and Schabus (2013). However, the current results clearly demonstrate that compared with actigraphy, the automatic cEEGrid‐based classifiers perform significantly better in sleep–wake assessment, besides offering the opportunity to detect different sleep stages. Although it is known that actigraphy is not necessarily a reliable assessment of sleep, it is often chosen for convenience. Having an easily mounted EEG solution similar to the one proposed here promises a better trade‐off between accuracy and usability. Additionally, our automated approaches do not provide very accurate estimates of the latency to the first REM period. This is likely to be a result of the short duration of this period and leads to some very noisy statistics, as seen in Figure 6.

The current dataset is larger than previous ear‐EEG sleep studies. However, 15 subjects are still not a large sample size and further validation in larger cohorts will be beneficial before using the system in basic and applied sleep research. Given that most classifiers, including the one used here, perform better with large training sets (see Mikkelsen & de Vos, 2018), reliability of automated scoring might further improve with a larger dataset.

We emphasise that all participants in this study were healthy and good sleepers. However, although we did not attempt to identify the characteristics of common sleep disorders, we aimed to imitate realistic variability in sleep quality and patterns. This was implemented by keeping subjects in bed for approximately 12 hr. A wide range of sleep durations and sleep onset latencies were obtained as a consequence and we consider this a strength of this study.

Regarding future work, the results presented here need to be confirmed in clinical cohorts and also in healthy participants in older age categories before the cEEGrid can be considered as a replacement for PSG in a clinical and research setting.

Overall, the results of this study are encouraging, as automated scoring combined with easy‐to‐use EEG monitoring holds great promise for future sleep monitoring in a much wider population than currently possible.

## ACKNOWLEDGEMENTS

The authors KBM and MDV received a grant from Circadian Therapeutics to perform this study. Additionally, the research was further supported by the Wellcome Trust, SCNi grant 098461/Z/12/Z, EPSRC IAA grant EP/K503939/1 and the NIHR Oxford Biomedical Research Centre.

## CONFLICT OF INTERESTS

JKE, MABC, NS, VLR, SD, DJD and AS declare no conflicts of interest, including any involvement in organizations with a financial interest in the subject matter of the paper. KBM and MDV received a grant from Circadian Therapeutics to perform this study. MDV is a founding member of Circadian Therapeutics.

## AUTHOR CONTRIBUTIONS

KM performed data analysis of EEG and NS analysed the actigraphy. KM, MDV, DJD and AS wrote the manuscript. GA performed visual scoring of the EEG. MABC, NS, MDV, JE and VR performed the measurements. MDV, AS, DJD and SD designed the study. All authors approved the final version of the manuscript.

## Figures and Tables

**Figure 7:**
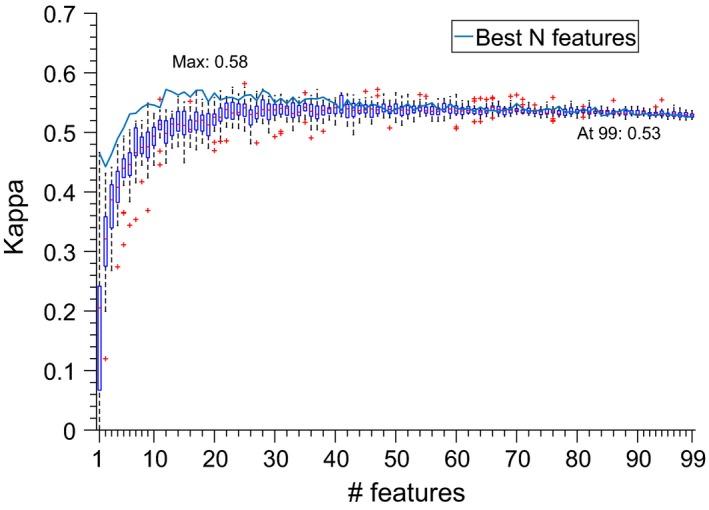
Analysis of classifier performance as a function of number of features. Box plots show results of random sampling from the feature space; solid line shows performance of N highest ranked features. We notice that the optimal number of features appears to be around 20–30, but also that the overfitting resulting from using the full set is relatively minor (given that the decrease in kappa is only from 0.58 to 0.53)

## APPENDIX 1

### RECORDING ALIGNMENT

As the clocks from the different wireless recording solutions could not easily be aligned, polysomnography (PSG) and cEEGrid recordings were aligned according to one of two approaches.

The primary approach (employed in 11 subjects) was based on the presence of large motion artifacts. It was designed in the following manner:

1. A single scalp derivation from the PSG recording (C3:M2) and a single electrode (L7, L8 or L5) from the cEEGrid recording (referenced to R4b) was extracted. The positive envelope of each signal was extracted.
2. For each envelope, the 90th percentile was calculated. All data points in each envelope below the 90th percentile were discarded (set to 0), resulting in a time series consisting of 90% zeros.
3. The cross‐correlation was calculated for the two time series. A clear peak in the cross‐correlation indicated the corresponding lag between the two measurements.

In four subjects, this approach resulted in multiple peaks in the cross‐correlation, meaning that the correct alignment could not be uniquely defined. In these cases, we instead relied on the slow‐wave portion of the electroencephalogram (EEG) in the following manner:

1. Data rejection, as described in Appendix 2, was performed on the cEEGrid recording.
2. The average of the right “C” was subtracted from the average of the left “C”, creating a purely lateral derivation. Likewise, the signal from the right mastoid was subtracted from the signal from the left mastoid.
3. Each of the two time series was filtered with a pass‐band of 0.3–4 Hz.
4. The filtered signals were rectified (meaning all values were exchanged for their absolute value).
5. The cross‐correlation was calculated between the two time series.

In all four cases, the cross‐correlation had a single dominant peak.

We chose to keep the first method in the 11 subjects for which it worked, because the corresponding cross‐correlation spectra were significantly cleaner. Hence, in the situations where it offered a well‐defined alignment, this was regarded as being the most probable (the two methods may differ in their estimates, in the order of less than a second).

## APPENDIX 2

### DATA REJECTION

Because the two different EEG set‐ups have different susceptibilities to both movement and electrode artefacts, automatic data rejection was performed before the recordings were fed into an automatic sleep scoring algorithm. This data rejection algorithm was fine‐tuned to match the results of a manual rejection performed on a single trial recording. The automatic rejection consisted of two steps:

1. The recording was partitioned into 2‐min epochs. For each channel in each epoch, the standard deviation was calculated. If the standard deviation exceeded 80 μV, the electrode was deemed faulty in the given time period and rejected.
2. After faulty channels were rejected individually, the recording was partitioned into 1‐s epochs with 50% overlap. For each epoch, power in the 5.7–54 Hz band was calculated. If the power exceeded 2.1·10^*−*12^ *V* ^2^ */*Hz for at least 14% of the electrodes present (excluding those rejected previously), the whole epoch was rejected.

After all epochs were marked, an additional two steps were taken to clean up the epoch rejections. (a) An epoch was only rejected if at least three consecutive epochs (meaning a 2‐s window) were rejected. (b) Long runs of rejected epochs, interspersed with short windows of accepted data, were merged, by also rejecting the intermediate epochs. Two rejected epochs were merged if their combined duration was at least five times as long as the separating gap.

## APPENDIX 3

### FEATURE SELECTION

We investigated feature selection in the following manner.

A classifier was trained using the entire dataset as test data. Using this classifier, we gained a ranking of all 99 features (based on the Gini coefficient, as supplied by the “ClassificationBaggedEnsemble” class in Matlab), depending on how well each of them separates the five classes. Using the ranking, we randomly drew N distinct features, letting the ranking weight the drawing (such that more important features are preferred). The drawing and weighting were carried out using the “datasample” function in Matlab. For each *N* ∈ (1*,* 99), 20 random drawings were performed. Additionally, two drawings were done in which the *N* best features were used (two drawings were necessary because of the variability inherent in the bagging algorithm). For each drawing, leave‐one‐out classifiers were trained and applied to all subjects, leading to kappa calculations.

Figure 7 shows both the distribution of kappa values coming from the random drawings and the averages from the two deterministic drawings. It can be seen that the optimal number of features is about 20–30, but also that the change between 99 and the optimal number is rather small, at maximum an improvement of 0.05 in kappa value. Because of this small benefit, we have chosen to keep the full set of features as this is a more straightforward approach.
